# Massive third wave of COVID-19 outbreak in Bangladesh: a co-epidemic of dengue might worsen the situation

**DOI:** 10.2217/fvl-2021-0182

**Published:** 2022-03-08

**Authors:** Popy Devnath, Md. Jamal Hossain, Talha Bin Emran, Saikat Mitra

**Affiliations:** ^1^Department of Microbiology, Faculty of Sciences, Noakhali Science & Technology University, Noakhali, 3814, Bangladesh; ^2^Department of Pharmacy, State University of Bangladesh, 77 Satmasjid Road, Dhanmondi, Dhaka, 1205, Bangladesh; ^3^Department of Pharmacy, BGC Trust University Bangladesh, Chittagong, 4381, Bangladesh; ^4^Department of Pharmacy, Faculty of Pharmacy, University of Dhaka, Dhaka, 1000, Bangladesh

**Keywords:** Bangladesh, COVID-19 pandemic, dengue burden, SARS-CoV-2, SARS-CoV-2 Delta variant, third wave

COVID-19 caused by a highly pathogenic etiological agent, SARS-CoV-2, has imposed massive burdens on overall healthcare and public health systems worldwide, especially in low- and low-middle-income countries, including Bangladesh [1,2]. Moreover, death tolls associated with other diseases, such as vector-borne diseases, have become overlooked and the resources available to address the outbreaks of non-COVID-19 diseases are insufficient in many areas due to the oversized burden associated with COVID-19 patient management. WHO works with partners to provide education and improve public awareness, so that people know how to protect themselves and their communities from mosquitoes, ticks, bugs, flies and other vectors [3,4].

## The massive third wave of COVID-19 outbreak in Bangladesh

The sudden surge of cases caused by the newly identified SARS-CoV-2 double mutant Delta variant (B.1.617.2) in the neighboring country India, has collapsed its healthcare and public health system; however, a mass vaccination program has proven effective, with new daily cases and deaths drastically reducing during recent days [5]. All signs indicate that Bangladesh is on the brink of experiencing a third COVID-19 wave due to the emergence of this new variant. Earlier, in April 2021, an increase in new cases was defined as a second wave, and a country-wide lockdown proved to be effective in slowing virus transmission during this period [6]. Besides, commencing a fruitful mass vaccination during February 2021 might be a determinant for the early ending of the second wave of COVID-19 in Bangladesh, though the country suffered from vaccine shortages from April 2021. However, in July 2021, daily cases were rising again due to the highly infectious Delta variant of concern having exceptional transmissibility. According to the Institute of Epidemiology, Disease Control and Research (IEDCR), the Delta variant of SARS-CoV-2 was first identified in Bangladesh on 8 May 2021, and it became the dominant strain in June 2021, identified in 78% of the total positive samples sequenced during the month [5,7]. This strain also manifested infections in 20–55% of the people who have previously recovered from COVID-19 caused by the other variants. Besides, several epidemiological and *in vitro* data demonstrated that the variant exerted approximately eight-fold reduced sensitivity to Oxford-AstraZeneca and Pfizer-BioNTech vaccine-generated immunity compared with the alpha variant [8].

As expected, the COVID-19 death toll has been rising since the start of July 2021, and there has been more than 200 associated deaths in a single day for the first time ever in Bangladesh [9]. The vaccination rate in Bangladesh is still lower compared with other countries, with only 27% of the population becoming fully vaccinated; whereas, around 40% of the Indian population (neighboring country of Bangladesh) received both doses of COVID-19 vaccines as of 17 December 2021 [10]. In addition to the rising number of cases associated with the Delta and omicron (B.1.1529) variants, another cause of high mortality and morbidity observed in COVID-19 patients is the onset of mucormycosis, caused by a black fungus belonging to the order Mucorales [5,11]. The first reported black fungus case was also on 8 May 2021, with a second case reported on 23 May 2021. According to the Directorate General of Health Services (DGHS), one death has been confirmed due to mucormycosis [5,12].

## Dengue situation in Bangladesh

Dengue is the most prevalent vector-borne viral disease causing a critical flu-like sickness and sometimes precipitating a likely lethal complexity designated as severe dengue. Commonly transmitted by *Aedes* mosquitoes, an estimated 3.9 billion people in over 129 countries are at risk of contracting dengue, which is associated with an estimated 40,000 deaths every year [13]. Approximately, 100 million global dengue cases have been reported yearly, with the highest number of cases being reported from the Asia–Pacific region [14,15]. The overall incidence of dengue appears to have increased 30-fold over the past 50 years [16]. Along with daily COVID-19 cases, seasonal changes have affected the overall number of dengue cases. An analysis of approximately 40,000 dengue cases over a 17-year period, from 2000 to 2017, indicated that about 50% of all dengue cases are reported during the monsoon season (May–August), whereas the remaining 50% cases are reported during the post-monsoon season (September–December) in Bangladesh [17]. In Bangladesh, annual outbreaks of dengue have been very common since 2000, and in 2019 the country faced the worst outbreak of dengue to date [18]. During 2019 the country reported 101,354 dengue cases associated with 179 deaths, according to information from the IEDCR and DGSH of Bangladesh [19]. Repeated dengue outbreaks have emerged as a serious public health threat for the country in terms of both morbidity and mortality.

## COVID-19 & dengue in Bangladesh: dual attacks & associated risk factors

The combined burden of newly increasing dengue cases and the increase in COVID-19 infections caused by the Delta variant have the potential to exacerbate an already stressed healthcare system in Bangladesh. Earlier, a multicenter assessment study warned that dengue and natural disasters could worsen the COVID-19 situation in Bangladesh [20]. In addition, the first combined case of COVID-19 and dengue in Bangladesh was reported on 15 May 2020 [21]. Thus far, the health authorities have reported approximately 28,177 dengue cases and 103 confirmed dengue-related deaths until 19 December 2021 [22]. The DGHS, on the other hand, has not released statistics on proven co-infection cases till now.

Co-infection with both SARS-CoV-2 and dengue virus in a patient poses a serious challenge for accurate diagnosis and the proper treatment of both viral diseases [23]. The initial symptoms associated with both viruses include fever, aches and rash, and may be difficult to differentiate, especially in a co-infected patient [24], and these viruses also present with similar diagnostic features such as thrombocytopenia, and in many cases anti-DENV antibodies cross reactivity with SARS-CoV-2 antigens lead to misdiagnosis of COVID-19 as dengue fever [24,25].

High population density is the primary reason for the rapid transmission of both dengue and COVID-19. The capital city of Bangladesh is Dhaka, one of the most populated cities in the world. Dhaka has experienced high numbers of dengue cases between 2012 and 2019. Since 2016, many dengue endemic countries approved the use of the first-licensed dengue vaccine (CYD-TDV); however, reports of more severe disease manifestations in seronegative individuals interrupt the implementation plan of dengue vaccine in Bangladesh [26], and mosquito control with adulticides and larvicides remains the primary effective prevention strategy [17]. The simultaneous outbreak of two vulnerable diseases in a tropical or subtropical country would represent a catastrophic event. In the year 2021, the number of dengue patients was 43 in May, but the number increased to 225 in June, and in October there was approximately 23,357 dengue cases according to DGHS [22].

It is evident that an alarming upward trend has been observed in dengue infections during the last couple of months in Bangladesh. The bidirectional attacks mediated by the dengue epidemic and the massive third wave of COVID-19 have aggravated Bangladesh’s already vulnerable healthcare system [24]. However, all the struggles and attempts of the country are now acting to combat the existing devastating COVID-19 pandemic [27]; in contrast, the emerging viral-borne dengue outbreak is not being focused on. Besides, the national monitoring of daily dengue cases are not updated regularly on the DGHS website which had been updated last in February 2021 [28] and it might be extremely likely that there is abundant under reporting.

The gravity of this co-epidemic should be underlined and the situation must not be handled with laxity by the authorities, especially as we did not observe countrywide vigorous campaigns against dengue fever, targeting both the common people and healthcare providers. Furthermore, if the upsurge of dengue cases in Bangladesh is equivalent to COVID-19, it would be unrealistic to provide health services and treatment to patients suffering from both infections.

## Conclusion & future perspective

The concurrence of COVID-19 and dengue outbreaks requires urgent attention to avoid further disaster. Both public health and socioeconomic conditions will be highly disrupted if cases of both viral infections continue to increase in the near future. Efforts should be taken to prevent and control vector-borne viral spread. In the present circumstances, the first recommended step is the immediate implementation of mosquito-control measures, as the start of the monsoon season has already placed a significant burden on COVID-19 and dengue management. During this season, community participation and public awareness of mosquito-control activities should be strengthened. In addition to these measures, increased awareness at the diagnostic level must be promoted to improve the identification of both COVID-19 and dengue co-infection and ensure that physicians are aware of the possibility of co-infections. Furthermore, the major strategy for limiting dengue is the control of the mosquito vector, and the death toll can be minimized by ensuring the early diagnosis of both diseases. Overall, the mass campaigns and practical approaches that strive for immediate and steady identification and supportive remedial control of dengue and COVID-19 cases are influential factors that urgently need to be executed.
